# Capecitabine in combination with docetaxel and mitomycin C in patients with pre-treated tumours: results of an extended phase-I trial

**DOI:** 10.1038/sj.bjc.6604067

**Published:** 2007-11-13

**Authors:** T Ernst, K Merx, U Gnad-Vogt, N Lukan, M Kripp, B Schultheis, A Hochhaus, R-D Hofheinz

**Affiliations:** 1 Onkologisches Zentrum, III. Medizinische Klinik, Medizinische Fakultät Mannheim der Universität Heidelberg, Theodor-Kutzer-Ufer 1-3, Mannheim 68167, Germany

**Keywords:** capecitabine, docetaxel, mitomycin, advanced cancer

## Abstract

Preclinical data suggest that the anti-tumour activity of capecitabine is enhanced by taxanes and mitomycin C through up-regulation of thymidine phosphorylase (TP). Here, we studied safety and efficacy of the combination of capecitabine with docetaxel and mitomycin C. Two dose levels (DL) were investigated: capecitabine 1000 mg m^−2^ b.i.d. on days 1–14, docetaxel 40 mg m^−2^ i.v. day 1, mitomycin C 4 or 6 mg m^−2^ i.v. day 1 (DL I or II). Cycles were repeated every 3 weeks. The primary aim was to determine the dose-limiting toxicities (DLT) during the first two treatment cycles and the maximum tolerated dose (MTD). A total of 46 patients (pts) refractory to standard therapies were enrolled, of whom the majority had gastrointestinal tumours (*n*=40). 14 pts had received ⩾3 lines of prior chemotherapy. At DL I, one out of six pts experienced DLT. At DL II, two out of six pts had DLT (mucositis grade 3). Thus, DL I was determined as MTD. Among a total of 37 pts treated on DL I the following toxicities were observed during cycles 1 and 2 (number of patients with grade 1/2/3/4 toxicity; NCI-CTC v. 3.0): anaemia 10/8/3/0, leucocytopenia 4/11/1/2, thrombocytopenia 0/1/2/0, diarrhoea 8/1/2/0, stomatitis/mucositis 3/3/1/0, nausea/vomiting 10/2/0/0, and hand-foot skin reaction 5/1/1/0. Of 30 pts who received at least two treatment cycles nine achieved complete or partial remissions, six pts achieved minor remissions, and seven pts had stable disease (tumour control rate 73%). Of note, four out of 10 patients with pancreatic cancer had partial remissions. In conclusion, capecitabine can safely be combined with docetaxel (40 mg m^−2^) and mitomycin C (4 mg m^−2^). The established regimen was well tolerated and the preliminary efficacy data in this heavily pre-treated patients population appears to be promising.

To date, no standard recommendations for salvage therapies exist for most types of gastrointestinal tumours with the exception of colorectal cancer. A benefit of salvage therapy has clearly been demonstrated in this disease in three clinical trials comparing either irinotecan, panitumumab or cetuximab against best supportive care (Cunningham *et al*, 1998; Au *et al*, 2007; van Cutsem *et al*, 2007). Randomized trials addressing the question of salvage treatment in other gastrointestinal cancers have not been published so far, and the question how to treat patients with gastrointestinal cancer once beyond the control of first line therapy is unanswered.

Capecitabine, an orally administered fluoropyrimidine carbamate produces high concentrations of 5-fluorouracil (5-FU) preferentially in tumour tissue. It is converted to its active metabolite 5-FU through a three-step enzymatic process, the final step of which is mediated by the enzyme thymidine phosphorylase (TP) (Schuller *et al*, 2000). Among various cytostatics studied in combination with capecitabine in human cancer xenografts, the taxanes and mitomycin C had the highest potential of increasing the activity of intratumoural TP (Sawada *et al*, 1998). Thus, in an attempt to increase the cytotoxic potential of capecitabine, several studies were conducted using capecitabine in combination with one of these TP inductors.

In a previous trial we established a combination regimen of capecitabine and mitomycin C which showed a favourable safety profile and promising activity in patients with pre-treated gastrointestinal cancer (Hofheinz *et al*, 2004). Phase-II studies have confirmed the encouraging activity of this combination with an acceptable toxicity profile in patients with pre-treated (Chong *et al*, 2005) or chemotherapy-naïve (Rao *et al*, 2004) advanced colorectal cancer. In a randomised phase-II trial comparing mitomycin C with either capecitabine or gemcitabine in patients with advanced biliary tract cancer, the mitomycin C plus capecitabine combination was superior in terms of response rate, progression-free survival and overall survival (Kornek *et al*, 2004). Capecitabine plus mitomycin C has been demonstrated to be an effective and well tolerated treatment option even in advanced breast cancer patients resistant to anthracyclines and taxanes (Maisano *et al*, 2005; Massacesi *et al*, 2006).

The clinical impact of up-regulating TP has further been demonstrated for capecitabine in combination with docetaxel in a variety of human malignancies in phase-I studies (Pronk *et al*, 2000; Nadella *et al*, 2002). In a large phase-III trial the addition of capecitabine to docetaxel resulted in a significant superior survival over single-agent docetaxel in 511 anthracycline-pretreated patients with advanced breast cancer (O'Shaughnessy *et al*, 2002). This randomised trial revealed that the combination of both drugs provided clear benefits over single-agent docetaxel in terms of objective tumour response rate (42 vs 30%), time to progression (median, 6.1 vs 4.2 months), and overall survival (median, 14.5 vs 11.5 months). Furthermore, several phase-II studies on the combination of capecitabine and docetaxel showed activity in patients with metastatic gastric cancer (Chun *et al*, 2005; Giordano *et al*, 2006), metastatic oesophageal cancer (Lorenzen *et al*, 2005), advanced non-small cell lung cancer (Han *et al*, 2003; Kindwall-Keller *et al*, 2005), and prostate cancer (Morant *et al*, 2004; Ferrero *et al*, 2006).

With the present trial, we sought to establish a three-drug regimen combining capecitabine with both of the preclinical most potent TP inductors, docetaxel and mitomycin C. The primary aim of this clinical trial was to determine the dose-limiting toxicities and the recommended dose of 3-weekly docetaxel and mitomycin C in combination with standard capecitabine in patients with pre-treated tumours.

## PATIENTS AND METHODS

The study protocol was reviewed and approved by the local institutional review board and the study was performed according to the Declaration of Helsinki. All patients provided written informed consent prior to entry into the study.

### Eligibility criteria

Patients with histological confirmed locally advanced or metastatic cancer refractory to or relapsed after at least one palliative chemotherapy regimen were eligible for entry into the study. Additional inclusion criteria comprised: Eastern Cooperative Oncology Group (ECOG) performance status ⩽2, age ⩾18 years, life expectancy ⩾3 months, measurable disease, left ventricular ejection fraction (LVEF) >50%, cardiac insufficiency NYHA <2, adequate bone marrow function (leucocyte count ⩾3000 *μ*l^−1^, platelet count ⩾100 000 *μ*l^−1^), and adequate renal (serum creatinine ⩽1.4 mg dl^−1^ or creatinine clearance >60 ml min^−1^) and hepatic function (bilirubin ⩽2 mg dl^−1^).

### Staging procedures

Before study admission, all patients underwent a complete history, physical examination, ECG and chest X-rays. Cardiac ultrasonography was carried out to determine the LVEF. A full blood count with differential and serum chemistry was obtained within 14 days prior to the start of treatment. Weekly blood counts were obtained and serum chemistry repeated every third week or whenever clinically indicated. Assessment of LVEF was repeated before the initiation of every other treatment cycle. Computed tomography (CT) scans of the tumour-bearing region were performed within four weeks prior to the start of study treatment. Indicator lesions were assessed every six weeks.

### Treatment schedule and dose escalation

Capecitabine was administered orally within 30 min after a meal (generally after breakfast and evening meal) at a dose of 1000 mg m^−2^ b.i.d. on days 1−14 for all dose levels (DL). At DL I and II docetaxel was administered at a dose of 40 mg m^−2^ and mitomycin C was given as an i.v. bolus of 4 mg m^−2^ (DL I) or 6 mg m^−2^ (DL II) on day 1, respectively. At DL III and IV mitomycin C was scheduled at a fixed dose of 6 mg m^−2^, and docetaxel was administered at a dose of 50 mg m^−2^ (DL III) or 60 mg m^−2^ (DL IV) on day 1. Cycles were repeated every three weeks. Dexamethasone 8 mg was added intravenously prior to docetaxel/mitomycin C infusion, and all patients received standard antiemetic prophylaxis with 5-HT_3_ antagonists to avoid any bias relating to gastrointestinal toxicities. A maximum cumulative dose of mitomycin C was not defined in the study protocol. Nevertheless, a thorough monitoring for parameters of haemolysis/haemolytic-uraemic syndrome was foreseen by the study protocol.

Dose-limiting toxicities (DLT) during the first two cycles of chemotherapy were defined by the occurrence of one of the following toxicities: grade 4 leucocytopenia/neutropenia or thrombocytopenia, symptomatic thrombocytopenia (haemorrhage), grade 3 or 4 febrile neutropenia or any⩾grade 3 non-haematological toxicity except nausea/vomiting. At least three patients were enrolled per dose level, with this number being increased to six if DLT occurred in one patient. Dose escalation was halted if DLT occurred in two or more patients. The maximum tolerated dose (MTD) was defined as the highest dose at which not more than one out of six patients experienced DLT during the first and second course of chemotherapy (i.e. days 1–42). Individual dose escalation was not allowed. In order to adequately determine the safety of this combination, the recruitment of further patients at the MTD was foreseen by the study protocol.

### Safety and efficacy analyses

Adverse events were recorded weekly and graded according to the National Cancer Institute Common Toxicity Criteria (NCI-CTC, version 3.0). In terms of efficacy, complete response (CR), partial response (PR), minor response (MR), stable disease (SD) and progressive disease (PD) were defined according to WHO criteria. Survival was defined as the time between the start of chemotherapy and death, according to the Kaplan−Meier method (Kaplan and Maier, 1958). Progression-free survival was defined as the time between the start of chemotherapy and clinical signs of progressive disease.

### Statistical considerations

For confirmation of the safety of this new regimen the recruitment of further patients at the recommended dose level was foreseen. The frequency of patients experiencing DLT during this expanded study phase was required to be lower than 17% (i.e. about one out of six of the patients experiencing DLT). With a total of 32 patients, a rate of at least 83% of the patients experiencing no DLT during cycles one and two can be excluded using Simon's two-stage design with a power of 80% and an *α*-value of 20% if less than five out of 15 (first stage) or ⩽eight out of 32 patients (second stage) experienced DLT.

## RESULTS

### Patients' characteristics

A total of 46 patients were enrolled at our institution between July 2004 and January 2007. All patients were evaluable for safety. Characteristics of enrolled patients are described in Table 1. The majority of patients (72%) had an ECOG status ⩽1. Pancreatic cancer (28%), biliary tract cancer (22%), colorectal and oesophageal cancer (15%, each) were the most common malignancies. The liver (74%) and lymph nodes (41%) were the most common sites of metastases, followed by the lung (30%) and bone (17%). All patients with oesophageal and biliary tract cancers had measurable disease.

Patients had received a median of one line (range 1–6) of prior palliative chemotherapy (14 patients with ⩾3 lines) and 25 patients (54%) had received prior 5-FU-based treatment.

### Determination of the MTD and dose intensity

One of the first six patients at DL I had DLT (diarrhoea grade 3) and consequently DL II was opened. At DL II nine patients were included. Of these, two patients were not evaluable due to rapid clinical deterioration. Another patient had a non-toxicity-related treatment break of 2,5 weeks between the first and second treatment cycle and was not amenable for the determination of DLT. Two out of the first six evaluable patients included at DL II had DLT (mucositis grade 3) and three patients had leucocytopenia grade 3. Thus, DL I was determined as MTD, and as the recommended dose level for the recruitment of further patients.

Thirty-seven patients were enrolled at dose level I. These patients received a total of 157 cycles (median 4, range 1–13) of chemotherapy. In all, only five patients treated at this DL had DLT during the first four cycles: leucocytopenia grade 4 with mucositis grade 3, leucocytopenia grade 4, mucositis grade 3, hand-foot syndrome grade 3 with diarrhoea grade 3, diarrhoea grade 3, *n*=1 each. Eight patients stopped treatment after cycle one for the following reasons: DLT (leucocytopenia grade 4, *n*=1; hand-foot syndrome grade 3 with diarrhoea grade 3, *n*=1), worsening of general condition (*n*=3), death due to tumour progression (*n*=2), consent withdrawn (*n*=1). At dose level I, 29 out of 37 patients received a minimum of two complete cycles. Of these, 20 patients received at least four cycles, and 15 had five or more cycles of therapy.

Nine patients were enrolled at dose level II. These patients received a total of 32 cycles (media*n*=2, range 1–14) of chemotherapy. Four patients stopped treatment after one cycle for the following reasons: DLT (mucositis grade 3, *n*=2), death due to tumour progression (*n*=1), worsening of general condition (*n*=1). Five patients received a minimum of two complete cycles, three of whom received at least four cycles and two patients five or more cycles of therapy.

Dose intensity was calculated for patients treated at the recommended DL I who received at least two complete cycles of chemotherapy (29/37 patients). A median dose intensity of 100% was reached during cycles one and two for each drug. The relative mean values with respect to cycles one and two were as follows: capecitabine 96%, mitomycin and docetaxel 100%, respectively. With respect to cycles three and four, the values were as follows (median/mean; *n*=20 patients): capecitabine 100/96, docetaxel 100/98, mitomycin C 100/99.

Postponement of treatment during cycles one and two was required in three patients only with a mean delay of 20 days (range, 14–24 days). Two of the 20 patients receiving a third or fourth cycle of therapy had a treatment delay with a mean delay of 11 days (range, 7–14 days).

At the recommended dose level I, the mean cumulative dose of mitomycin C was 31 mg (median 28 mg; range 6–91 mg). 13 of 37 patients (35%) received more than 36 mg of mitomycin C and two patients received more than 76 mg.

### Safety

Table 2 summarises haematological and non-haematological adverse events during cycles one and two (the time period for the determination of DLT, i.e. days 1–42) and cycles three and four. At DL I, a total of 37 patients were evaluable for safety. Anaemia (*n*=21), leucocytopenia (*n*=18), fatigue (*n*=16), nausea/vomiting (*n*=12) and diarrhoea (*n*=11) were the most commonly observed adverse events during cycles one and two. Leucocytopenia grade 4 was observed in two patients (5%). Grade 3 toxicities were recorded in nine patients (24%): anaemia (*n*=3), thrombocytopenia (*n*=2), diarrhoea (*n*=2), leucocytopenia, mucositis, hand-foot skin reaction, fatigue (*n*=1, each).

During cycles three and four (*n*=23 patients), anaemia (*n*=15), hand-foot syndrome (*n*=10) and leucocytopenia (*n*=9) were the most commonly observed adverse events.

No evidence of mitomycin C-induced haemolytic-uraemic syndrome or pulmonary toxicity was documented.

### Anti-tumour activity

30 patients received at least two cycles of treatment and were evaluable for response analysis. One patient with oesophageal and one with biliary tract cancer achieved a complete remission, respectively. Seven patients achieved partial remissions (pancreatic cancer *n*=4, biliary tract cancer *n*=1, oesophageal cancer *n*=1, head and neck tumour *n*=1), six patients achieved minor responses (pancreatic cancer *n*=4, biliary tract cancer *n*=1, oesophageal cancer *n*=1), and seven patients had stable disease. Anti-tumour activity is summarized in Table 3. Thus, in total 15/30 (50%) achieved any kind of response and 7/30 (23%) had stable disease adding to a tumour control rate of 73%. Only 8/30 (27%) had progressive disease after cycle 2 while receiving therapy. Remarkably, all of four patients with colorectal cancer had a progressive disease.

The median progression-free survival for all patients (*n*=46) was 3.2 months (range, 0.2–15.8+), for patients with any kind of tumour response or stable disease (*n*=22) 5.0 months (1.7–15.8+). The duration of the complete remissions observed were 15.8+ months (biliary tract cancer) and 5 months (oesophageal cancer). The median survival for all patients was 5.2 months (0.5–17.1+), for patients with tumour response or stable disease 8.2 months (2.1–17.1+), for patients with progressive disease 4.5 months (2.4–7.2+).

## DISCUSSION

Docetaxel as well as mitomycin C caused an up-regulation of thymidine phosphorylase (TP) activity and an enhancement of capecitabine efficacy in a human xenograft model (Sawada *et al*, 1998). TP activity is frequently induced in human cancers and its induction protects cells from apoptosis and stimulates angiogenesis (Toi *et al*, 2005). Since TP-targeting treatment has been developed in parallel with analyses of biological functions and translational research, therapies targeting this enzyme might be a rationale chemotherapeutic approach. Combinations between TP-inducible treatments and TP-targeting therapies seem to have improved response and survival in patients with various types of cancer, e.g. breast cancer (O'Shaughnessy *et al*, 2002). In the present extended phase-I trial we sought to combine capecitabine, a TP-targeting therapy with the two most potent TP-inducing chemotherapeutics, docetaxel and mitomycin C.

Our current findings indicate that capecitabine 1000 mg m^−2^ orally b.i.d. on days 1–14 in combination with docetaxel 40 mg m^−2^ and mitomycin C 4 mg m^−2^ i.v. day 1 every 3 weeks is both effective and well tolerated. Moreover, this regimen appears to be convenient using i.v. application in 3-weekly intervals. Dose escalation in this phase I study continued only to DL II. Due to two patients suffering DLT (mucositis grade 3) at this dose level, DL I was determined as MTD. Haematological adverse events, predominantly anaemia and leucocytopenia were the most commonly observed adverse events in a total of 37 patients treated at the MTD. Twenty patients received at least four cycles of chemotherapy on DL I and the toxicity profile observed during the following cycles was comparable. Only hand-foot skin reaction was more frequent in cycles three and four. The median dose intensity was maintained at 100% during the first four cycles for each drug. In addition, there were no episodes of haemolytic uraemic syndrome or pulmonary toxicities during the study.

The preliminary activity (response rate 30%, tumour control rate 73%) observed in 30 patients evaluable for efficacy appeared to be promising in view of the high number of patients with previous 5-FU exposure and the high rate of heavily pre-treated patients in this study. Of note, these results were obtained with comparably low doses of three-weekly docetaxel (40 mg m^−2^) and mitomycin C (4 mg m^−2^). These findings underline our hypothesis that the clinical activity might even be increased by the combination of two instead of one TP-inductor(s) with the TP-targeting agent capecitabine. Of course, these results need confirmation by phase-II trials in distinct tumour entities.

Three-drug regimens containing capecitabine and mitomycin C have not been reported, while several three-drug combinations containing capecitabine and docetaxel have been published. Capecitabine, docetaxel and gemcitabine were investigated in a phase-I trial in various solid tumours by Amarantidis and coworkers (Amarantidis *et al*, 2006). The toxicity in 18 patients was described to be generally mild, and responses were seen in some patients. Thirty-five patients with metastatic pancreatic cancer (66% of them chemotherapy-naïve) received this three-drug regimen in a recently published study (Fine *et al*, 2008). A comparably high rate of tumour remissions (29%) was reported, but the rate of grade 3–4 thrombocytopenia (14%) was higher than in the present trial (5%). Other investigators added cisplatin in a variety of solid tumours (Fakih *et al*, 2005), carboplatin in metastatic oesophagogastric cancer (Tsai *et al*, 2005) or epirubicin in patients with advanced breast cancer (Venturini *et al*, 2003) to the combination of capecitabine and docetaxel. Particularly the phase-II study of Venturini *et al* described promising anti-tumour activity in a total of 67 patients with chemotherapy-naïve metastastic breast cancer (response rate 82%). Nevertheless, a total of 16% of the patients had febrile neutropenia.

In conclusion, we have established a three-drug dosing schedule for the combination of capecitabine, docetaxel, and mitomycin C that is well tolerated and showed encouraging signs of activity especially in patients with pancreatic, biliary tract and oesophageal cancer. This regimen may be considered for patients after failure of pre-treatment but it is also likely to be a valuable, cost-saving and convenient treatment option even in earlier stages of cancer chemotherapy.

## Figures and Tables

**Table 1:** Patient demographics

**Variable**	**No. of patients (%)**
*Enrolled*	46
Dose Level 1	37 (80)
Dose Level 2	9 (20)
	
*Age (years)*	
Median	63
Range	43–78
	
*Gender*	
Male	29 (63)
Female	17 (37)
	
*Performance status (ECOG)*	
Median	1
0	8 (18)
1	25 (54)
2	13 (28)
	
*Primary tumour site*	
Pancreatic cancer	13 (28)
Biliary tract cancer	10 (22)
Colorectal cancer	7 (15)
Oesophageal cancer	7 (15)
Cancer of unknown primary	3 (7)
Head and neck tumour	3 (7)
Gastric cancer	1 (2)
Hepatocellular carcinoma	1 (2)
Small intestinal cancer	1 (2)
	
*Prior chemotherapy*	
1 Line	25 (54)
2 Lines	7 (15)
⩾3 Lines	14 (31)
5-FU based (i.v. or oral)	25 (54)
	
*Tumour localisation*	
Primary	19 (41)
Liver	34 (74)
Lung	14 (30)
Lymph nodes	19 (41)
Bone	8 (17)
Peritoneal carcinomatosis	5 (11)

**Table 2:** Haematological and non-haematological adverse events per dose level in cycles 1/2 and 3/4 (worst per patient)

**No. of patients/NCI-CTC grade^a^**																
	**Dose level 1 (*n*=37)**								**Dose level 2 (*n*=9)**							
	**Grade 1**		**Grade 2**		**Grade 3**		**Grade 4**		**Grade 1**		**Grade 2**		**Grade 3**		**Grade 4**	
**Cycles**	**1/2**	**3/4**	**1/2**	**3/4**	**1/2**	**3/4**	**1/2**	**3/4**	**1/2**	**3/4**	**1/2**	**3/4**	**1/2**	**3/4**	**1/2**	**3/4**
*Anaemia*	10	9	8	6	3	—	—	—	4	1	3	2	—	—	—	—
*Leucocytopenia*	4	—	11	7	1	2	2	—	1	1	1	—	3	2	—	—
*Thrombocytopenia*	—	2	1	—	2	—	—	—	—	—	1	—	—	—	—	—
*Nausea/vomiting*	10	4	2	—	—	—	—	—	3	—	1	—	—	—	—	—
*Stomatitis/mucositis*	3	3	3	2	1	1	—	—	—	—	—	—	2	—	—	—
*Diarrhoea*	8	2	1	3	2	—	—	—	—	1	—	—	—	—	—	—
*Hand-foot skin reactions*	5	7	1	3	1	—	—	—	—	—	—	—	—	—	—	—
*Alopecia*	5	5	3	2	—	—	—	—	—	—	2	1	—	—	—	—
*Fatigue*	11	3	4	4	1	—	—	—	3	—	—	—	—	—	—	—
*Polyneuropathy*	7	3	—	—	—	—	—	—	—	—	—	—	—	—	—	—
*Renal function (creatinine)*	2	2	2	1	—	—	—	—	—	—	—	—	—	—	—	—

a Graded according to the National Cancer Institute Common Toxicity Criteria (version 3.0).

**Table 3:** Anti-tumour activity in patients who received a minimum of two cycles of chemotherapy

**Tumour type**	***n***	**CR**	**PR**	**MR**	**SD**	**PD**
Pancreatic cancer	10	0	4	4	2	0
Biliary tract cancer	7	1	1	1	2	2
Oesophageal cancer	6	1	1	1	2	1
Colorectal cancer	4	0	0	0	0	4
Others	3	0	1	0	1	1

CR=complete remission; PR=partial remission; MR=minor remission; SD=stable disease; PD=progressive disease.
